# Nanoparticles for Glioblastoma Treatment

**DOI:** 10.3390/pharmaceutics17060688

**Published:** 2025-05-23

**Authors:** Dorota Bartusik-Aebisher, Kacper Rogóż, David Aebisher

**Affiliations:** 1 Department of Biochemistry and General Chemistry, Collegium Medicum, Faculty of Medicine, University of Rzeszów, 35-310 Rzeszów, Poland; dbartusikaebisher@ur.edu.pl; 2 Student Scientific Club English Division, Collegium Medicum, Faculty of Medicine, University of Rzeszów, 35-310 Rzeszów, Poland; kr117626@stud.ur.edu.pl; 3 Department of Photomedicine and General Chemistry, Collegium Medicum, Faculty of Medicine, University of Rzeszów, 35-310 Rzeszów, Poland

**Keywords:** glioblastoma, nanoparticles, radiosensitization, targeted therapy

## Abstract

GBM is the most common and aggressive primary brain tumor in adults, characterized by low survival rates, high recurrence, and resistance to conventional therapies. Traditional diagnostic and therapeutic methods remain limited due to the difficulty in permeating the blood–brain barrier (BBB), diffuse tumor cell infiltration, and tumor heterogeneity. In recent years, nano-based technologies have emerged as innovative approaches for the detection and treatment of GBM. A wide variety of nanocarriers, including dendrimers, liposomes, metallic nanoparticles, carbon nanotubes, carbon dots, extracellular vesicles, and many more demonstrate the ability to cross the BBB, precisely deliver therapeutic agents, and enhance the effects of radiotherapy and immunotherapy. Surface functionalization, peptide modification, and cell membrane coating improve the targeting capabilities of nanostructures toward GBM cells and enable the exploitation of their photothermal, magnetic, and optical properties. Furthermore, the development of miRNA nanosponge systems offers the simultaneous inhibition of multiple tumor growth mechanisms and the modulation of the immunosuppressive tumor microenvironment. This article presents current advancements in nanotechnology for GBM, with a particular focus on the characteristics and advantages of specific groups of nanoparticles, including their role in radiosensitization.

## 1. Introduction

Glioblastoma multiforme (GBM) is the most common primary malignant brain tumor of glial origin [1]. The annual incidence of GBM in the U.S. is estimated at 3.22/100,000 people, and it is more common in men; moreover, its risk increases with age—the peak incidence of low-grade gliomas is between the ages of 35 and 45, and high-grade gliomas peak in the seventh and eighth decades of life [2,3]. The overall 5-year survival rate from diagnosis is only about 6.8%, with an average survival of only 15 months [2,4]. A known risk factor is exposure of the head and neck region to ionizing radiation, while a history of atopic diseases such as asthma, atopic dermatitis, or inhalant allergies is considered a protective factor [2]. Glioblastoma multiforme accounts for nearly 50% of all CNS malignant tumors. The other 50% includes diffusely infiltrating lower-grade gliomas, primary central nervous system (CNS) lymphoma (7%), and malignant forms of ependymomas (3%) and meningiomas (2%) [5]. GBM is detected using MRI with gadolinium contrast, and a biopsy with histopathological and molecular evaluation of the tumor is required to confirm the diagnosis [5]. Based on these, tumor grade and features are also assessed, which is necessary for treatment planning. Molecular studies provide a better understanding of the GB microenvironment, including genomic, epigenomic, transcriptomic, and proteomic characteristics, as well as its interactions with the immune system, which has applications in the development of new therapy techniques [6]. Symptoms of brain tumors are varied and often uncharacteristic and may result from increased intracranial pressure or the compression of brain structures by the tumor mass [3]. Symptoms that should alarm the physician include the progressive deterioration of higher neurological functions; new or progressive headache, especially in the morning, combined with nausea and vomiting; papilledema on fundus examination; seizures; and visual field abnormalities [3]. There are many therapeutic methods for GBM. Traditional methods include surgery, chemotherapy, and radiation therapy—the first-line treatment of glioblastoma multiforme includes surgical resection, followed by radiation therapy and alkylating chemotherapy with temozolomide [5]. Of course, the choice of method depends on the stage of the tumor, the presence or absence of metastases, the clinical condition, and the patient’s comorbidities, especially since the above methods have many side effects, including the destruction of healthy cells in addition to cancerous ones in the case of chemotherapy and radiotherapy. In the case of chemotherapy, a common obstacle is the limited effect of chemotherapeutics on GBM due to the need for drugs to penetrate the blood–brain barrier [7]. In addition, the tumor is often characterized by resistance to radiotherapy. Thus, the development of new therapeutic methods that are more selective and have as little systemic toxicity as possible is being pursued. Targeted methods that exploit differences in receptor expression on the surface of tumor cells allow drugs to selectively bind to them, sparing healthy cells [7]. Modified monoclonal antibodies such as Bevacizumab, in turn, can attach to enzymes or growth factors necessary for cancer cell growth, such as vascular endothelial growth factor (VEGF), thereby inhibiting neovascularization and tumor expansion [8]. Glioma immunotherapy, on the other hand, focuses on stimulating the immune system to “set it up” to fight diseased cells, including through cancer vaccines, as well as by affecting the tumor microenvironment (TME) [7]. Another of the targeted immunotherapy approaches is the use of immune checkpoint inhibitors, such as anti-CTLA-4 (e.g., ipilimumab) and anti-PD-1/PD-L1 (e.g., nivolumab, pembrolizumab), which increase the population of T cells beneficial for destroying cancer cells, as well as the invention of modified CAR-T T cells, which recognize tumor antigens and activate upon contact with them, leading to the destruction of pathological cells [9,10]. Another alternative way to treat GBM is PDT, photodynamic therapy, which is a minimally invasive treatment method that uses photosensitizers that selectively accumulate in tumor tissue and light, under which they become activated. The result is the generation of reactive oxygen species and the destruction of the pathological cell [7,11]. PDT is a dynamically developing method using new generations of photosensitizers, which are in many cases based on nanoparticles, such as natural-product-based, carrier-free, noncovalent NPs with excellent synergistic anticancer activity or NanoVP (nanoverteporfin), which shows a 2-fold increase in photosensitizer uptake and superior PDT efficacy in GBM cells compared to liposomal VP [12,13]. These and other targeted therapies increase selectivity in destroying tumor cells but still have some limitations, related, for example, to difficulty in permeating the blood–brain barrier or resistance to therapy, related, for example, to the differential expression of receptors on tumor cells (such as PD-1), which may translate into the ineffective binding of monoclonal antibodies and reduce the therapeutic effect [14]. Another problem is the immunosuppressive specificity of the GBM microenvironment, which limits the effectiveness of immunotherapy, including the action of CAR-T lymphocytes, as well as the high heterogeneity of the tumor, as manifested by the multiplicity of its phenotypes [15]. To overcome these and other difficulties, the development of nanotechnology has been gaining importance in recent years. This broad field of science presents a variety of solutions that can significantly enhance the effectiveness of both standard and targeted GBM therapies. Nanoparticle upgrades such as surface functionalization, peptide modifications, and cell membrane coating increase the specificity of nanocarriers toward GBM cells and enhance their photothermal, magnetic, and optical properties [16]. To increase the penetration capability of BBB, nanocarriers based on lipids, polymers, metals, or dendrimers as well as carbon nanotubes and dots are used [16]. To increase the efficiency of mRNA-based immunotherapy, including mRNA vaccines, small mRNA-loaded extracellular vesicles (sEVs) have been created that penetrate the BBB and to which antibodies that selectively target the cancer cell attach [17]. Nanosponges, in turn, promote the uptake of multiple miRNAs involved in growth, migration, invasion, angiogenesis, and the formation of the tumor’s immunosuppressive microenvironment, thereby inhibiting GBM progression [18]. A variety of nanoparticles can also serve as potential radiosensitizers, enhancing radiation-induced damage in GBM cells through various mechanisms [19]. In the following article, we will try to present current developments in the field of nanotechnology applied to the treatment of glioblastoma multiforme, enhancing the efficacy of both standard treatments such as chemotherapy and radiotherapy as well as immunotherapy and other targeted therapies. The use of nanotechnology in the aspect of GBM therapy is a relatively new phenomenon, and the vast majority of the NPs described in this review have not yet been incorporated into clinical trials, and the results of the studies presented are mainly on cell lines or animal models. Our work aims to highlight the importance of a diverse group of nanoparticles as a modern method of supporting the therapy of an extremely aggressive cancer such as glioblastoma multiforme by presenting their unique features and mechanisms of action and pointing the way forward. The inclusion of nanoparticles in clinical trials is a key step in assessing their safety and long-term effectiveness.

This article is a review of the literature available in the PubMed database. The authors of this review worked on the basis of an agreed scheme, selecting articles based on their title, language, abstract, and access. The original search term was “Nano-Based Technology for Glioblastoma”. 45 results were received. The search criteria considered were: time of publication of the article—2020–2025, language of publication-English or Polish, and species-humans (search status as of 6 April 2025). Then the search phrases “Nanoparticles AND Glioblastoma” and different configurations of the described nanoparticles with “Glioblastoma” were searched, such as “Nanoliposomes AND Glioblastoma”, “Dendrimers AND Glioblastoma”, “Gold nanoparticles AND Glioblastoma”, “Metal oxide nanoparticles AND Glioblastoma”, “Nanotubules AND Glioblastoma” and so on. Phrases on the role of nanoparticles in GBM radiosensitization and immunotherapy were also searched. Only articles published within the last 5 years [2020–2025] were considered. 

Inclusion criteria:

- qualifying articles relating to the use of nanoparticles in the treatment of GBM
- qualifying articles relating to the use of nanoparticles in the radiosensitization of GBM
- qualifying both in vivo and in vitro studies
- qualifying both abstracts and full text articles

Exclusion criteria:

- articles in a language other than English or Polish
- articles from before 2020
- articles with content that does not correspond to the subject of the article
- articles no clearly defining effect of nanoparticles on GBM

## 2. Nanoparticles: General Characteristics

**Figure 1:**
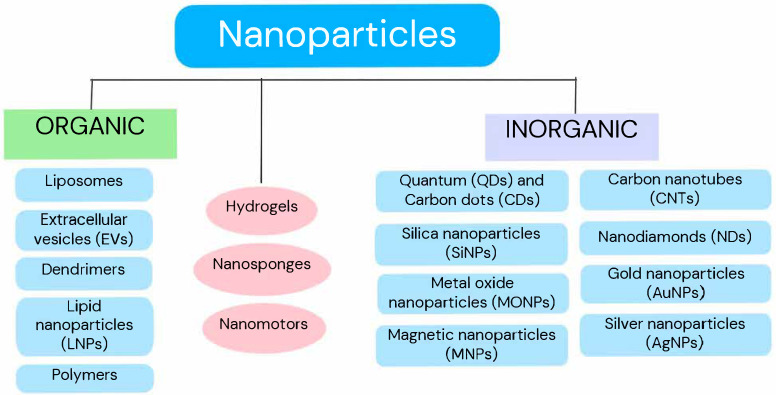
General division of the nanoparticle groups discussed in this review.

Nanoparticles are particles between 1 and 100 nanometers in size. Through their large surface-to-volume ratio, they can exhibit different physical and chemical properties compared to larger materials. Their properties also depend on the shape or type of surface, which offers the possibility for numerous modifications depending on their purpose [19]. Most notably, their small size gives them the ability to penetrate the BBB (this is possible for particles up to about 300 nm), which will be crucial for their use in treating CNS cancers [20]. Nanoparticles (NPs) act as nanocarriers capable of carrying large amounts of drug to the target site with controlled release. They can easily penetrate the finest capillaries and avoid phagocytic clearance, thus extending the half-life in plasma and ensuring sustained drug release. NPs are used to deliver biomolecules such as proteins, peptides, enzymes, genes, and vaccines. Their properties are used in anticancer therapy, gene therapy, immunotherapy, tissue regeneration, metabolic disorders, neurological disorders, and theranostics [21,22]. Depending on the material, nanoparticles can be formed from organic matter, e.g., liposomes, dendrimers, polymers, and lipid nanoparticles (NLPs), including solid NLPs; inorganic matter, e.g., nanoparticles of metal oxides, gold, silver, nanodiamonds, carbon dots, and nanotubes; or a combination of two or more materials [19]. Nanoparticles can serve as carriers for drugs, chemotherapeutics, or monoclonal antibodies that selectively bind to receptors on the surface of cancer cells and also contain other ligands on their surface, such as folic acid, hyaluronic acid, arginylglycyl-aspartate tripeptide (RGD), or epidermal growth factor (EGF). With the latter, however, there is a risk of less selectivity and NP attachment to healthy tissue as well [23]. Nanoparticles can act as a hydrogel, particularly effective in penetrating the BBB [24]. Biomimetic nanoparticles can also act as a carrier for mRNA, creating an anticancer vaccine that easily integrates into the tumor cell due to its structure mimicking biological membranes [25]. The multiplicity and diversity of nanoparticle carrier materials and forms, as well as the continuous progress in creating new ones, contribute to this field’s rapid development and responsiveness to emerging difficulties and needs. (Figure 1). On the other hand, the potential adverse effects of nanoparticles should also be mentioned. Nanoparticles can penetrate cells and cause oxidative stress, DNA damage, and activate inflammatory response pathways, such as Titanium dioxide nanoparticles, which induced inflammation in both in vitro and in vivo models. The risk of NPs accumulation in tissues and organ toxicity (liver, lung, brain) is also not excluded. NPs can also trigger immune reactions, including allergies and autoimmunity. For instance, the use of carbon nanotubes caused allergic airway inflammation in mouse models. Long-term exposure can lead to genotoxicity and even cancer-e.g., chronic exposure to carbon nanotubes can lead to mesothelioma. A key aspect of safety is biodegradability - nanoparticles that decompose into non-toxic compounds are considered safer than permanent, metallic or carbon forms [24]. 

## 3. Organic Nanoparticles

### 3.1. Polymers

The first type of nanoparticles we are discussing are polymers. They have a variety of physicochemical properties that can be tailored to the desired applications. The surface of polymeric materials can be modified by the introduction of various functional groups: hydroxyl (-OH), carboxyl (-COOH), or amine (-NH2) allow the nanoparticle to interact with biomolecules such as proteins or nucleic acids, enabling the specific adsorption or immobilization of these molecules on their surface. Polymeric materials are also relatively inexpensive [26]. Nanopolymers include chitosan, alginate, heparin, polylactide, polyacrylamide, and polycaprolactone. Special attention is currently being paid to chitosan, which is a mucopolysaccharide of natural origin, closely related to cellulose. It is biodegradable, biocompatible, stable, non-toxic, and can undergo chemical or enzymatic modifications [21]. In an article by Ramalho, Maria João et al. [27], nanoparticles made of poly(lactic-co-glycolic acid) carrying the chemotherapeutic gemcitabine, coated with chitosan, were created and administered to the tumor through the nasal cavity. In vivo and in vitro studies on GBM cell lines confirmed that the created nanoparticles were characterized by adequate mucoadhesiveness for this mode of drug delivery and efficiency in its selective release in the tumor mass. Thus, it was proven that the developed molecule with the use of chitosan CH-GEM-NPs is an effective tool to support the effectiveness of GBM chemotherapy. Nanopolymers can be used to better visualize and ablate GBM, increasing its susceptibility to phototherapy, as in the work of Su, Xiang et al. [28], where by modifying the side chains and substituents in the phenothiazine and thiophene groupings, an NIR-II polymeric luminogen with high fluorescence quality, good solubility, excellent photothermal conversion, and balanced reactive oxygen species generation was obtained, maximizing the use of photons in synergistic photodynamic/photothermal therapy in drug-resistant glioma. Similarly, a paper by He, Yichen et al. [29] describes a second near-infrared (NIR-II) absorptive polymer (PDTP-TBZ) conjugated with intense electron donor dithienopyrrole (DTP) and strong electron acceptor thiadiazolobenzotriazole (TBZ). The nanoparticle thus created, after laser irradiation in in vitro and in vivo experiments, showed high phototoxicity against cancer cells while exhibiting biosafety. On the other hand, a paper by Hsu, Fei-Ting et al. [30] presented glycopolymer-like condensed nanoparticles (∼60 nm) (GC NPs) that induced cellular reactive oxygen species (ROS), leading to tumor cell death and M2 to M1 macrophage polarization, thereby reducing the acidic tumor microenvironment and immunosuppression. In GBM models, the nanoparticles remodeled the tumor microenvironment from “cold” to “hot”, enhancing the efficacy of anti-PD-L1/anti-PD-1 therapy by promoting macrophage polarization and activating cytotoxic T lymphocytes (CTL) and dendritic cells (DC).

### 3.2. Liposomes

Another subgroup of organic nanoparticles is liposomes. They represent some of the most advanced nanocarriers used in drug delivery, including for the treatment of glioblastoma multiforme (GBM) [31]. They are spherical vesicles composed of one or more concentric lipid layers surrounding an aqueous interior. Due to their structure, liposomes can transport both hydrophobic substances (located in the lipid layer) and hydrophilic substances (located in the aqueous core). The number of bilayers determines the amount of drug that can be transported in them [32]. In addition, it is possible to load them with other bioactive molecules, such as peptides, proteins, RNA, and DNA [33,34]. Due to their small size (2.5 to 25 nm), they are able to efficiently penetrate the BBB [32]. A key advantage of liposomes is their high biocompatibility and ability to modify surfaces. For example, coating liposomes with polyethylene glycol (PEG) creates a “shield” that protects them from recognition by the reticuloendothelial system, which significantly increases circulation time in the blood and reduces immunogenicity [35]. Moreover, the dense PEG coating promotes better penetration across the blood–brain barrier (BBB) and into the brain parenchyma [35,36]. An interesting method of drug release from the PEG-coated lysosome is the reaction of IgG and IgM anti-PEG antibodies with the nanoparticle membrane, as reported in a study by Chen, Even et al. [36]. As a result of the formation of the attacking immune complex, the membrane of the lysosome is ruptured and doxorubicin is released. This method of premature drug release is applicable to patients who have previously developed antibodies against PEG. In the context of GBM, liposomes allow for the targeted delivery of anticancer drugs directly to the tumor focus, minimizing toxic effects on healthy tissues. Appropriately designed liposomes can also be equipped with targeting ligands, such as antibody fragments or molecules that recognize receptors overexpressed in cancer cells. Such targeting increases the efficacy of the therapy while reducing the risk of side effects [33,34]. The transport of liposomes across the BBB can take place in three ways: transporter-dependent cellular transfer (TMT), adsorption (adsorption-mediated transfer), and receptor-mediated transfer (RMCT) [37]. Of particular relevance here are interactions with glutathione transporter proteins and the transferrin receptor (TfR), which are commonly present in brain endothelial cells (BECs) [37]. Currently, there are clinical trials on liposomes for the treatment of GBM, such as rhenium nano-liposomes (186RNL) NCT01906385, RNA-LP vaccine NCT04573140, and liposomal curcumin (LC)NCT05768919. A brief description of these nanoparticles and the purpose of the studies is shown in Table 1. Currently recruiting trials were selected.

### 3.3. Dendrimers

Dendrimers represent one of the most advanced classes of organic nanocarriers used in therapeutics (GBMs). They are branched symmetric molecules with precisely controlled structures that can be chemically modified to enhance therapeutic efficacy and targeted drug delivery [33]. Due to their unique architecture and physicochemical properties, dendrimers exhibit a high capacity to penetrate the blood–brain barrier (BBB) and accumulate within the brain tumor. They can serve as carriers of anticancer drugs and kinase inhibitors as well as genetic material (e.g., siRNA) [33]. Of particular importance in the treatment of GBM are dendrimers like PAMAM, PLL, and PPI, which—with or without targeting ligands—can effectively transport drugs into the tumor. Surface modifications using sugars (e.g., galactose, mannose), peptides (e.g., angiopep-2), or molecules that recognize receptors overexpressed on tumor cells (e.g., LRP1, TfR) enable them to specifically target glioma cells, microglia, or tumor-associated macrophages (TAMs) [38]. Dendrimers can also act as carriers in immunomodulatory therapy—for example, by the targeted delivery of CSF-1R receptor inhibitors (such as BLZ945), which promotes the repolarization of the tumor immune environment and improves the anti-tumor response. Furthermore, dendrimer systems have shown efficacy in eliminating cancer stem cells, which may be crucial in preventing disease recurrence. In a study by Knauer, Nadezhda et al. [39], researchers validated a polycationic phosphorus dendrimer carrying siRNA into cancer cells with high expression of Lyn kinase. They noted that the introduction of anti-Lyn siRNA resulted in a significant reduction in tumor cell viability, which was not observed for cells without Lyn expression. The effect of the dendrimer on the expression of markers such as PD-L1, TIM-3, and CD47 on the surface of GBM cells has also been described, which can be used in cancer immunotherapy. For dendrimers, intranasal application is also possible for direct drug delivery to the brain, bypassing the BBB, thus minimizing systemic effects and limiting toxicity [40]. Such a mode of delivery with positive results was described in a study by Mignani, Serge et al. [40], where dendrimers were used to transport drugs such as haloperidol, paeonol, dextran, insulin, and calcitonin. Dendrimers can also act as contrast agents for brain imaging, competing with the nephrotoxic gadolinium, as outlined by Wu, Yufei et al. [41]. Well-water-soluble dendrimers consisting of up to 192 TEMPO radical units have been created, and in mouse studies, they showed good biocompatibility and selectivity in reaching GBM cells, which is a promising direction in the development of brain tumor imaging techniques [41].

### 3.4. Extracellular Vesicles EVs

Extracellular vehicles (EVs) represent a diverse population of nanostructures that naturally play a role in intercellular communication. Their major fractions include exosomes, which, due to their size (<200 nm) and unique molecular composition, show significant potential for diagnostic and therapeutic applications [42]. Exosomes are formed by separating from cell membranes and contain various concentrations of proteins, lipids, and cholesterol in their structure [33]. Unlike most manufactured nanoparticles, exosomes can immediately bind to receptors on individual target cells. In contrast, factors that can be challenging in the use of EVs include their proper biostabilization, the efficient loading of drugs into exosomes, and their removal from circulation [43]. In the context of glioblastoma multiforme (GBM), EVs enable the efficient transfer of biological molecules—such as miRNAs, proteins, or lipids—into tumor cells. They can be used both in monotherapy, where they exhibit immunomodeling and anti-inflammatory effects, as well as in combination with other anticancer treatments [43]. In a study by Hong, Soohyun et al. [44], EVs carrying miRNAs (miR-124 EVs) were applied to human GBM cell lines and microglia, resulting in an anti-tumor effect through tumor growth suppression and an immunogenic effect through the activation of NK cells in the tumor microenvironment. Exosomes isolated from 3D cultures of GBM cells, after modification, show the ability to inhibit the proliferation, migration, and invasion of glioma cells [45]. Moreover, the use of surface engineering techniques allows EVs to target selected cell receptors, which increases their specificity against cancer cells. An example is the development of immunogenic exosomes (imsEVs) carrying mRNA for IFN-γ and presenting anti-CD71 and anti-PD-L1 antibodies on their surface, which have demonstrated anti-tumor efficacy even against tumors resistant to immunotherapy [45]. EVs can also target the enzymes and growth factors necessary for tumor cell division, inactivating them [33]. Intracellular vesicles, taken from the microenvironment of GBM cells, can further serve diagnostic purposes as cancer biomarkers. After analyzing their composition, including genetic material as well as membrane proteins, one can learn more about the tumor itself. Integrated “all-in-one nanowire assay” systems can be used to capture and analyze cancerous EVs [46]. In a study by Salviano-Silva, Amanda et al. [47], EVs taken from the blood of patients with newly diagnosed and recurrent GBM were examined using imaging flow cytometry for the presence of eight glioma-associated antigens and tetraspanins and compared with those from healthy individuals. EVs with Tenascin-C (TNC) present were found in GBM patients and high levels of double-positive TNC+/CD9+ EVs were found in newly diagnosed GBM. High levels of TNC expression are found in malignant glioma cells, especially in cells with mesenchymal features and chromosomal aberrations. This study demonstrated the feasibility of using the analysis of EVs from patient serum as tumor biomarkers, also useful for identifying tumor-specific mutations in liquid biopsies.

### 3.5. Lipid Nanoparticles

Lipid nanoparticles (LNPs) are some of the most intensively researched drug delivery systems, showing high efficiency in transporting both hydrophilic and hydrophobic therapeutic molecules [48]. Their primary advantage is their ability to enhance intracellular drug uptake by immune and tumor cells, which is particularly important in the context of the activation of endosomal TLR receptors (including TLR3, TLR7/8, TLR9) and cytoplasmic receptors such as RIG-I and MDA-5 [49]. Solid lipid nanoparticles (SLNs), which are one class of LNPs, combine numerous advantages—they are biocompatible, biodegradable, exhibit low toxicity, improve drug solubility, and provide the controlled release of active substances. Due to their physicochemical properties and high cellular penetration ability, they are an attractive alternative to traditional colloidal and polymeric carriers [48,50]. Nanoparticles can be administered directly to the tumor by intratumor injection or during surgical resection, allowing high drug concentrations to be achieved at the lesion site [50]. Currently, LNPs are being intensively developed as non-viral carriers for gene therapies, using mRNA, siRNA, pDNA, and antisense oligonucleotides (ASOs) [51]. Their composition is usually based on ionizable lipids, cholesterol, PEG lipids, and structural lipids [52]. LNPs enable the efficient transfection of components of the CRISPR-Cas9 system in GBM cells in vitro, and one contemporary work by Rouatbi, Nadia et al. [51] also demonstrated the possibility of efficient gene editing in vivo in orthotopic GBM models using LNPs containing CRISPR RNA. Thus, this study demonstrates the feasibility of using CRISPR-Cas9 LNPs as a potential tool for in vivo screening in refractory glioblastoma [51]. LNPs are also gaining importance as carriers in combination therapies such as photodynamic therapy (PDT). Combining natural photosensitizers (e.g., curcumin or parietin) with LNPs enables the precise delivery of these molecules to GBM cells, increasing therapeutic efficacy while reducing side effects [53]. In addition, innovative LNP platforms containing superparamagnetic iron oxide nanoparticles and proapoptotic drugs such as nutlin-3a have been designed to induce hyperthermia and tumor apoptosis. By functionalizing the molecules with the peptide angiopep-2, these systems show the ability to selectively transport across the BBB and target drug delivery to glioma cells [54]. In addition, LNPs can serve to translocate chemotherapeutics such as doxorubicin, topotecan, paclitaxel and SN38, gemcitabine, temozolomide, and vincristine into GBM cells, serving to improve their pharmacokinetic profile and increase the selectivity of action [55].

## 4. Inorganic Nanoparticles

### 4.1. Carbon Nanotubes

Carbon nanotubes (CNTs) and especially single-walled carbon nanotubes (SWCNTs) are considered very promising nanomaterials in medicine and biotechnology. Their ability to penetrate biological membranes, their ability to functionalize, and their potential for targeted drug transport make them attractive tools for both diagnostics (as imaging probes) and anticancer therapies, including the treatment of glioblastoma multiforme (GBM) [56]. Carbon nanotubes can effectively transport therapeutic molecules across the blood–brain barrier (BBB), extend the circulation time of drugs in the body, and enable their targeted delivery through chemical binding to ligands. However, despite these advantages, the use of CNTs in medicine faces significant limitations related to their toxicity and lack of biodegradability [57]. Long-term studies in cell lines and animal models have shown that carbon nanotubes can induce immunosuppressive responses by downregulating the expression of key surface glycoproteins such as HLA-DRA and HLA-DRB1 as well as disrupt genomic stability by deregulating lamina B expression, indicating their potential genotoxic effects [55,58]. In the context of brain tumors, carbon nanomaterials are finding application as a component of extracellular matrix engineering in cancer cell migration studies. One example is three-dimensional polymer scaffolds (NFS), whose stiffness is modified by the introduction of multi-walled carbon nanotubes (MWCNTs) [59]

### 4.2. Carbon and Quantum Dots

Quantum dots (QDs) are semiconductor nanoparticles with unique optical properties that are distinguished by their narrow emission spectrum, wide excitation range, high quantum yield, and significant photostability. Thanks to these characteristics, QDs have gained considerable interest in medicine, especially as tools for simultaneous imaging, biomarker detection, and drug delivery. The extensive surface functionalization capabilities of QDs allow for their precise targeting, making them useful in cancer therapy—especially difficult-to-treat brain tumors such as glioblastoma multiforme (GBM) [60,61]. One promising research direction is the use of quantum dots for image-guided therapy. One example is the construction of glioma-specific QDs-c(RGDyk)NP nanoparticles, which were combined with glioma-targeted cyclopeptide c(RGDyk) and poloxamer-188. Through synergy with ultrasound-targeted microbubble destruction (UTMD) techniques, these particles have demonstrated the ability to specifically accumulate in glioma tissue in rats, enabling the precise surgical removal of the tumor while maintaining safety [62]. Modern approaches also include the use of QDs in direct therapy—as in the case of photosensitizer-free laser therapy (PS-free-LT) using a 1267 nm laser, where QDs play a key role in activating apoptosis, inhibiting tumor cell proliferation, and improving lymphatic outflow [63]. Moreover, the use of QDs as tracers in polymeric nanocarriers (e.g., PLGA NPs modified with poloxamer 188) allows for the tracking of biodistribution and optimizing the delivery of anticancer drugs to the brain, which is crucial for effective GBM therapy [64]. Despite their impressive potential, classical QDs containing heavy metals (e.g., CdSe) raise toxicological concerns, especially in the context of neurological applications. Therefore, research on alternative structures, such as graphene quantum dots (GQDs) and carbon quantum dots (CDs), which exhibit higher biocompatibility and biostability, is being intensively developed. GQDs, thanks to their ability to penetrate the blood–brain barrier, can not only deliver drugs to glioma cells but also show a synergistic effect with classical chemotherapy, increasing cell membrane permeability and therapeutic efficacy [65]. Carbon dots (CDs) are a modern class of carbon nanomaterials less than 10 nm in diameter, combining the features of classical semiconductor quantum dots (QDs), such as a wide excitation spectrum, tunable fluorescence, and high photostability, with the advantages of carbon materials—good biocompatibility, non-toxicity, and easy chemical functionalization [65,66]. In anticancer applications, CDs show great potential both as drug carriers and as standalone therapeutic agents. One example is Cu-doped carbon dots (Cu-CDs), with extended excitation times. This allows for the efficient generation of reactive oxygen species (ROS) in response to low-frequency ultrasound—a mechanism that forms the basis of innovative sonodynamic therapy (SDT). Importantly, Cu-CDs induce the process of “cuproptosis”—a specific form of cell death dependent on the presence of copper ions [67]. Moreover, in vitro studies have shown that the combination of CDs with the inhibitors of signaling pathways significantly increases the anti-tumor efficacy of the therapy by reducing the migration and invasion of tumor cells and lowering the levels of proteins responsible for cell survival and proliferation, such as Akt, Bcl-2, STAT3, and Slug [68]. A paper by Perini, Giordano et al. [61] reported on the synergistic effects of CDs together with the chemotherapeutic agent doxyrubicin on GBM cells, concluding that these nanoparticles show efficacy against tumor cells both by directly destabilizing the cell membrane and indirectly enhancing the efficacy of chemotherapeutic drugs. In addition to therapeutic applications, CDs are widely used as fluorescent probes in cell imaging and biomolecule sensors and as elements in catalysis, photocatalysis, and photosynthetic augmentation processes. However, despite significant advances in the synthesis of these nanomaterials, challenges remain in their reproducibility and the control of their chemical structure. Small changes in synthesis conditions can lead to significantly different final properties, and the mechanisms of CD formation and the relationship between structure and photoluminescence are not yet fully understood [65].

### 4.3. Magnetic NPs

Magnetic nanoparticles (MNPs) represent a promising theranostic platform, combining diagnostic and therapeutic capabilities in a single structure. Due to their unique physicochemical properties—such as their ability to be controlled by an external magnetic field, biodegradability, surface functionalization, and biological compatibility—MNPs are being widely studied as targeted drug delivery systems and imaging agents for the treatment of GBM [69]. MNPs exhibit the ability to penetrate the blood–brain barrier (BBB), making them uniquely suited for the treatment of CNS diseases. Their surface can be modified with targeting ligands, such as the angiopep-2 peptide, allowing active transcytosis and directional drug delivery to tumor cells via the LRP-1 receptor. MNPs can also be combined with exosomes and nucleic acids (e.g., siRNA) to form complex therapeutic platforms with high anti-tumor efficacy [70]. One of the most innovative applications of MNPs is synergistic therapy, including magnetic hyperthermia (MHT), a process in which MNPs are heated using an external electromagnetic field, leading to a local temperature rise above 42 °C and the induction of cancer cell death [71,72,73]. Combining MHT with chemotherapy (e.g., temozolomide—TMZ) significantly increases treatment efficacy while reducing systemic toxicity. This approach has been validated in animal models, where functionalized nanocarriers (Ang-TMZ-LMNVs) showed selective accumulation in tumor tissue, the inhibition of GBM cell invasion and proliferation, and the significant prolongation of survival [71]. A study by Pulvirenti, Luca et al. tested magnetic Fe_3_O_4_ NPs carrying TMZ into tumor cells. Combined with metal oxide and high porosity, the nanoparticle increased the drug loading capacity and absorption capabilities. The results obtained after 48 h of treatment showed that the hybrid nanoparticles inhibited the viability of human glioma cells much more effectively than the free drug. The integration of thermal, magnetic, transport, and diagnostic properties makes magnetic nanoparticles an extremely attractive tool in modern nanomedicine. Their capabilities include magnetic resonance imaging (MRI) and photoacoustic (PA) and near-infrared (NIR) imaging, as well as support for conventional radiotherapy and chemotherapy [69,74].

### 4.4. Metal Oxide NPs

Metal oxide nanoparticles (MONPs) are a diverse group of nanomaterials that exhibit unique physicochemical and biological properties that make them extremely attractive candidates for applications in modern oncology. Due to their multifunctionality, MONPs are capable of supporting multiple therapeutic strategies in the treatment of glioblastoma multiforme (GBM), including chemotherapy, radiation therapy, photothermal therapy, and immunotherapy [75]. Among the most widely studied MONPs are iron oxide nanoparticles (e.g., Fe_3_O_4_, γ-Fe_2_O_3_), titanium oxide (TiO_2_), zinc oxide (ZnO), lanthanum oxide (La_2_O_3_), and more complex structures such as molybdenum or tungsten oxides (e.g., POMs—polyoxometalates). These nanoparticles exhibit a number of mechanisms of anticancer action, including the generation of reactive oxygen species (ROS); the enhancement of the effects of ionizing radiation (as radiosensitizers); the catalytic release of metal ions supporting chemotherapy (e.g., Fe^2^⁺); the induction of oxidative stress, apoptosis, and autophagy; and the transport and controlled release of anticancer drugs [75,76,77,78]. The use of MONPs in GBM therapy also allows them to cross the blood–brain barrier (BBB), a significant advantage in the context of the limitations of traditional treatments. For example, lanthanum oxide (La_2_O_3_) effectively penetrates GBM cells after intravenous administration, where it exerts cytotoxic effects through mechanisms of apoptosis, DNA damage, and the activation of autophagic pathways. Similar effects have been observed for POMs, which induce oxidative stress and impair cell division [76]. Metal oxides also show potential for radiation therapy. Nanoplatforms such as MnO_2_/Pt@BSA have been designed to overcome the resistance of hypoxic GBM cells by producing oxygen and enhancing the generation of hydroxyl radicals (-OH), enhancing the effectiveness of ionizing radiation. Moreover, iron oxides in the form of SPIONs (superparamagnetic iron oxide nanoparticles) or FMX (ferumoxytol) act as contrast agents in MRI imaging and can participate in redox reactions, such as enhancing the cytotoxic effect of sodium ascorbate (P-AscH-) [79]. Despite their advantages, some MONPs, such as TiO_2_, may affect the integrity of the BBB and cause neuroinflammatory immune responses, which require further toxicological studies and safety evaluation. Therefore, although MONPs exhibit a broad spectrum of beneficial effects, their clinical use requires careful design, dose optimization, and the evaluation of long-term biocompatibility [75].

### 4.5. Silica NPs

Silica nanoparticles (SiNPs), in both non-porous and porous forms (porous silica nanoparticles, PSiNPs), are among the most promising nanomaterials for cancer diagnosis and therapy, including glioblastoma multiforme (GBM). Their optimal physicochemical properties—such as high biocompatibility, large specific surface area, ability to functionalize easily, and ability to penetrate the blood–brain barrier—make them ideal carriers for anticancer drugs and potential standalone therapeutic agents [80,81]. The mechanisms of anti-cancer action of SiNPs are diverse and dependent on particle size, dose, and cancer cell type. Studies have shown that SiNPs with diameters of 5–20 nm, used at concentrations ≥50 µg/mL, can induce the apoptosis or necrosis of GBM cells by increasing the levels of reactive oxygen species (ROS), leading to oxidative damage and the activation of cell death pathways. These effects show a strong dependence on the type of cancer cells and exposure conditions [82]. Porous silica nanoparticles (PSiNPs) are characterized by large pore volumes, drug-loading capabilities, and controlled drug release. Their degradation is promoted by elevated levels of ROS present in the tumor microenvironment, making them particularly useful as anticancer drug delivery systems. A significant achievement in this field is the release of C dots—functionalized PSiNPs nanoparticles for targeted cancer imaging and therapy—into phase II clinical trials [80]. The use of modified PSiNPs as drug carriers, such as docetaxel (DTX), has demonstrated therapeutic efficacy in temozolomide (TMZ)-resistant glioma models. PEGylated PSiNPs with an octyl group modification (C8-MSN) not only efficiently penetrate the brain tumor barrier (BBTB), but also show selective accumulation within the tumor, prolonging survival and reducing side effects compared to the free form of the drug [83]. In addition, silica nanocarriers enable the encapsulation of novel anticancer compounds, such as ruthenium complexes. The functionalization of nanoparticles with amino acids has been shown to increase their internalization by GBM cells (e.g., the U87 line), leading to enhanced apoptosis and higher anticancer efficacy compared to the free form of the drug [84]. Immune cell-based drug delivery systems are an interesting and innovative direction for the development of SiNP-based therapies. Neutrophils, as the most abundant leukocytes capable of penetrating hard-to-reach barriers, have been proposed as a form of “Trojan horse” in the delivery of nanoparticles to inflammatory and tumor sites. Studies on the interaction of SiNPs with neutrophils open up new therapeutic possibilities, especially in the context of GBM recurrence prevention and drug delivery to the tumor microenvironment [85].

### 4.6. Gold NPs

Gold nanoparticles (GNPs) represent one of the most promising classes of nanomaterials for GBM therapy. Their unique physicochemical properties, such as high surface area-to-volume ratio, low toxicity, ease of functionalization, and ability to penetrate the blood–brain barrier (BBB), make them excellent candidates both as drug carriers and compounds to enhance the efficacy of radiotherapy in combination therapy [86,87]. In the context of radiotherapy, GNPs exhibit the ability to increase the local radiation dose through strong photon absorption resulting from their high atomic number (Z = 79). Initially, the radiosensitization effect was thought to be due solely to this physical mechanism, but an increasing number of studies have emphasized the importance of chemical and biological mechanisms of action as well [88]. Numerous preclinical studies have shown that GNPs improve the efficacy of radiotherapy. For example, Au@DTDTPA(Gd) nanoparticles significantly enhanced the therapeutic effect in mice with glioma by inhibiting tumor cell invasiveness compared to radiation therapy alone [88]. PEGylation of GNPs, or their coating with polyethyleneglycol (PEG), increases their stability, avoids the immune system, and improves their intracellular localization. PEG-GNPs caused a higher number of DNA double-strand breaks and a significant enhancement of the immune response through increased expression of pro-inflammatory cytokines in GBM cell lines [86]. Ultrasmall doxorubicin-conjugated GNPs (AuTio-Dox-AF647s) have demonstrated the ability to efficiently penetrate the BBB and accumulate in GBM organoids, confirming their potential as drug carriers for testing anti-cancer therapies in organoid models [87]. The use of GNPs as a core of nanostructured spherical nucleic acids (SNAs) enabled the systemic delivery of siRNAs to GBM tumors in a phase 0 clinical trial, resulting in the suppression of the Bcl2L12 oncogene without side effects, which was confirmed in patients’ tumor tissues [89]. Another application of GNPs is gallate-conjugated nanoparticles (GA-GNPs), which exhibit potent anti-tumor activity and a radiosensitizing effect against U251 GBM cells, opening prospects for their use in cancer theranostics [90]. The use of GNPs as a component of hydrogels for local drug release is also an interesting approach. In an in vivo study, a hydrogel containing quisinostat and GNPs enabled image-guided radiotherapy while providing radiation-induced controlled drug release, which significantly reduced tumor growth in a mouse model [91].

### 4.7. Silver NPs

Silver nanoparticles (AgNPs) have gained great interest in recent years as an innovative therapeutic tool in medicine, mainly due to their antimicrobial and anticancer properties. AgNPs are distinguished by their broad spectrum of biological activities and their ability to be modified by green synthesis, using natural plant extracts [92,93]. The green synthesis of Ag/AgCl-NPs using Curcuma rotunda tuber extract and Ziziphus mauritiana fruit extract yielded particles with high selectivity against cancer cells, including glioblastoma stem cells (GSCs) and Ehrlich tumor cells (EACs). Importantly, no significant cytotoxicity was observed against normal cells [94]. Silver and platinum compounds in the form of dendritic nanostructures (AgPt-NPs) have shown potent antimicrobial activity against Gram-positive and Gram-negative strains as well as selective cytotoxic activity against glioma and melanoma cells, with no toxicity against fibroblasts within a certain concentration range [92]. Of particular interest are small-sized AgNPs (5 nm), which have shown potent anti-tumor effects against the U-87MG line, including by increasing reactive oxygen species (ROS) production and inhibiting the Sonic Hedgehog (SHH) signaling pathway, which can lead to reduced tumor proliferation [93]. Studies combining AgCl-NPs with temozolomide (TMZ), a standard drug used in GBM therapy, showed a synergistic effect—the nanoparticles potentiated the effects of TMZ, suggesting their potential as adjuvants in glioma therapy [95]. Experiments using cold atmospheric plasma (CAP) in combination with polyvinyl alcohol (PVA)-coated AgNPs showed potent cytotoxic effects (>100-fold greater than either therapy alone) against U373MG GBM cells. This effect was dependent on oxidative stress and increased the uptake and accumulation of AgNPs after exposure to CAP [96]. In another study, AgNPs obtained by green synthesis showed the ability to interact with the NOTCH2 gene, which is overexpressed in gliomas, indicating that AgNPs could be used in brain tumor therapy at the molecular level [97].

### 4.8. Nanodiamonds

Nanodiamonds (NDs) are unique carbon nanomaterials that are gaining increasing importance in brain tumor diagnosis and therapy due to their physicochemical properties, biocompatibility, and functionalization capabilities [33,98]. One of the main challenges in the treatment of brain tumors is the poor permeability across the blood–brain barrier (BBB) and the low specificity of targeting drugs to the tumor site. In response to this problem, a system of nanodiamonds coated with a photostable biopolymer, denatured bovine serum albumin (dcBSA), PEGylated and functionalized with the three-amino-acid peptide RGD (arginine-glycine-aspartic acid), which has affinity for the receptors present in the vascularization of tumors, was developed [33,98]. RGD-dcBSA-PEG-NDs modified in this way show high colloidal stability in various buffer solutions and the ability to overcome the BBB barrier in in vitro models. Importantly, in a mouse model of U-87MG glioma, these nanoparticles show selective accumulation at the tumor site after intravenous administration, enabling effective tumor imaging with minimal toxic effects on other organs [98]. In addition to diagnostic applications, nanodiamonds also show therapeutic potential. In particular, conjugates of doxorubicin with polyglycerol nanodiamonds (Nano-DOX) can be delivered to the tumor by GBM-associated macrophages. In the study, Nano-DOX was shown to effectively inhibit the activity of the STAT3/IL-6 pathway, which plays a key role in the reciprocal activation of glioma cells and astrocytes. This looped pathway leads to increased therapy resistance and the migration, invasion, and proliferation of tumor cells [99]. In addition, Nano-DOX can negate the side effects of temozolomide (TMZ) therapy by reducing STAT3 and IL-6 activation induced by this drug, an additional advantage of these nanoparticles [99].

## 5. Other Forms of Nanoparticle Supply

### 5.1. Hydrogels

Hydrogels are three-dimensional hydrated structures formed by the crosslinking (covalent or non-covalent) of hydrophilic natural or synthetic polymers in an aqueous environment [100]. The properties of hydrogels, such as modulated stiffness, viscoelasticity, and stability, enable their delivery to brain tissue and their use for controlled drug release. Thus, hydrogels are gaining applications in tissue engineering, cell culture, tissue regeneration, and as drug delivery vehicles [100]. Traditional hydrogels with free drugs have limited efficacy in the treatment of solid tumors due to poor drug penetration. Therefore, there is growing interest in hydrogel composites containing nanoparticles, which combine the advantages of both technologies—controlled release and enhanced therapeutic efficacy [101,102]. In the work of Liu, Rong et al. [101], a ferritin-based hydrogel (Dox@HFn Gel) containing doxorubicin and oxidized dextran (Dex-CHO) was developed. This hydrogel is injected into the tumor environment and exhibits long-term retention and gradual release of the drug. Through active transcytose transport and the induction of immunogenic cell death, this system induces a strong immune response and reduces the risk of recurrence and metastasis [101]. Another example is a hydrogel containing nanoparticles with paclitaxel (PTX) and temozolomide (TMZ), which has shown efficacy in delaying tumor recurrence after GBM resection in in vivo models [102]. Similarly, lipid nanocapsules (LNCs) loaded with gemcitabine and functionalized with NFL peptide enabled the targeted delivery of the drug to GBM cells remaining after surgery, significantly reducing recurrence [103]. Adding nanoparticles to a hydrogel matrix increases mechanical strength, improves the control of drug release, and introduces “smart” functions such as response to external stimuli (e.g., magnetic field, NIR light, pH, electric field) [104]. This makes it possible to design complex structures that respond dynamically to changes in the environment and deliver the drug in a precise and personalized manner. Hydrogels containing nanoparticles are also used in GBM imaging. For example, a hydrogel with the radio-sensitive drug quisinostat and gold nanoparticles (AuNP) enables the simultaneous treatment and computed tomography (CT) imaging of the tumor. Radiation induces the degradation of the hydrogel and the release of the drug, leading to the inhibition of tumor growth and increased treatment efficacy [105].

### 5.2. Nanosponges

Nanosponges are novel structures of high architectural complexity, capable of the binding and controlled release of multiple bioactive molecules. They are characterized by their high cargo capacity, biocompatibility, ability to have simultaneous gene and chemical activity, and resistance to enzymatic degradation, which make them extremely attractive for therapeutic applications [106,107]. Therapeutic strategies target oncogenic miRNAs (e.g., miR-21, miR-221) to suppress tumor-promoting pathways or restore tumor-suppressive miRNAs to inhibit malignancy. Nanosponge technology aims to inhibit tumor progression in glioblastoma (GBM) by capturing and neutralizing oncogenic miRNAs that drive cancer development, thereby disrupting key molecular pathways involved in tumor growth, invasion, angiogenesis, and immune evasion. In light of the growing role of microRNAs (miRs) in the pathogenesis of GBM, nanosponges capable of simultaneously capturing multiple oncogenic miRs represent a new breakthrough in therapy. The use of miR-nanosponges enables the targeted uptake of miR-9, miR-21, miR-215, and miR-221—key regulators of processes such as proliferation, invasion, angiogenesis, and immunosuppression—leading to the multidimensional inhibition of GBM progression [106]. BV2 cell membrane-coated miR-nanosponges (BM@miR-nanosponge) have been developed to enhance blood–brain barrier (BBB) penetration and target GBM cells. The membrane-coating of miR-nanosponges improves tropism to the tumor and enables more effective modulation of the immune microenvironment. This type of therapy significantly prolonged the survival of mice with GBM, surpassing the effectiveness of the standard drug temozolomide (TMZ) [106]. Another breakthrough is RNA nanosponges (RNSs), created using the rolling circle transcription (RCT) technique, which makes it possible to obtain programmable, multilayered RNA structures. Such nanosponges are capable of delivering therapeutic molecules as well as genes, enzymes, or drugs, and their unique structure provides protection from nucleases and long persistence in serum [107]. To overcome the problem of limited content release from nanografts, a CRISPR-Cas13a-based activation strategy was introduced, whose RNase activity can be precisely controlled by the presence of specific tumor markers (e.g., EGFRvIII). Hierarchical RNA nanococoons (RNCOs-D) have been designed for this purpose, a system in which the Cas13a enzyme is “discovered” only in the presence of conditions specific to the tumor environment (e.g., low pH or the presence of specific mRNA) [107]. RNCOs-D combines chemotherapy with gene therapy, using both drug release and the precise CRISPR-Cas13a-mediated silencing of EGFRvIII oncogene expression, resulting in a potent therapeutic effect in vitro and in vivo [106,107]. As with most nanoparticles for the treatment of GBM, the long-term effects of the therapy are unknown, including tissue toxicity and accumulation outside of tumor cells. Further research is needed and, most importantly, the initiation of clinical trials to test their safety and long-term efficacy.

### 5.3. Nanomotors

Nanomotors are advanced nanostructures capable of autonomous movement in response to specific chemical stimuli, making them extremely promising for the treatment of glioblastoma multiforme (GBM). Their unique ability for chemotaxis, i.e., targeted movement toward areas with high concentrations of reactive oxygen species (ROS) and nitric oxide synthase (iNOS), allows them to effectively reach the tumor microenvironment [108]. Utilizing enzymatic properties (peroxidase and catalase-like activity), some nanomotors, such as CuO@CaO_2_ NSs, can autonomously generate H_2_O_2_ and O_2_, which activates photothermal (PTT), photodynamic (PDT), and chemodynamic (CDT) therapies. Their ability to move autonomously and produce hydroxyl free radicals (-OH) enables more effective destruction of cancer cells [109]. Nanomotors can be further modified to enhance their ability to cross the blood–brain barrier (BBB). An example is modification with the polypeptide angiopep-2 (Ang), which recognizes brain endothelial cells and GBM cells, allowing efficient penetration into the tumor. Once inside the tumor cell, the nanomotors release the drug TLND (a lonidamine derivative that targets mitochondria), which disrupts the metabolic symbiosis of tumor cells and promotes their apoptosis [108]. In addition, as they move toward the tumor, nanomotors produce nitric oxide (NO), which acts as an inducer of immunogenic cell death (ICD). NO also interacts with blood vessels within the tumor, which enhances the transport of immune cells and further strengthens the body’s immune response [109]. Nanoparticles for GBM treatment currently being in clinical trials are presented in Table 2.

## 6. The Role of Nanotechnology in GBM Radiosensitization

**Figure 2:**
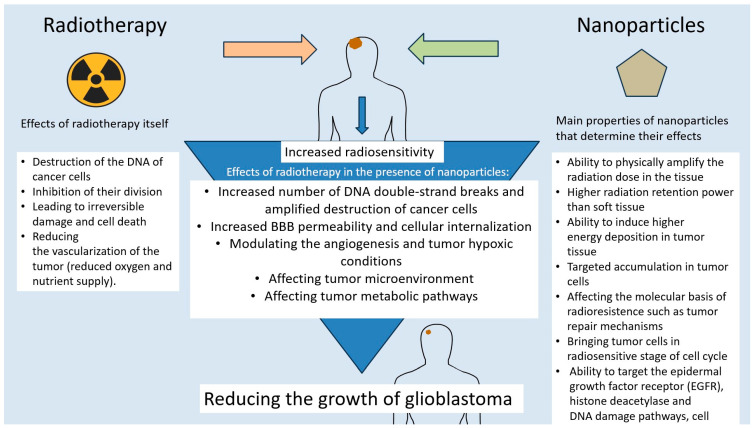
Scheme of the action of nanoparticles to increase the radiosensitivity of GBM.

Radiation therapy is one of the standard treatments for GBM, used at various stages of therapy, often as adjuvant or palliative treatment but also to complement radical surgery. The essence of radiation therapy is the destruction of cancer cells by ionizing radiation, directly and indirectly via free radicals. The goal of treatment is to deliver as much radiation as possible to destroy diseased cells, sparing healthy ones. Therefore, the maximum dose is determined by the toxicity to surrounding healthy tissues. Recent advances in radiation therapy techniques, such as intensity-modulated radiation therapy (IMRT), have greatly improved dose compliance and clinical outcomes by delivering multiple spatially modulated radiation fields [110]. Radioprotective drugs such as free radical scavengers, cell cycle regulators, inhibitors of radiation-induced apoptosis, and growth factors are being developed to enhance the protection of healthy tissues. In the case of GBM, the main difficulty in using RT lies in its frequent resistance to this type of therapy. This is due to a number of factors, including the heterogeneity of the tumor and its microenvironment, containing molecules and cells that promote its growth; hypoxia; changes in the tumor’s cellular metabolism that promote its survival and invasiveness; the presence of cancer stem cells, a subpopulation of cells within the tumor mass that have the ability to self-renew and differentiate into different types of cancer cells; the presence of various micro RNAs, modulating Akt signaling, cell-cycle checkpoint responses, and DDR activity or a disrupted cell cycle; and DNA repair processes and metabolic pathways such as PI3-kinase/Akt signaling pathway or JAK/STAT [111,112]. In response to GBM radioresistance, radiosensitizing drugs are being developed with a variety of modes of action, aimed at altering the activity of cellular factors, targeting the epidermal growth factor receptor (EGFR), histone deacetylase, DNA damage pathways, cell cycle regulators, cell death receptors, and modulating angiogenesis or tumor hypoxic conditions. Mechanisms of radiosensitization also include the inhibition of intracellular thiols, the formation of cytotoxic substances, the inhibition of repair biomolecules, and effects on tumor metabolic pathways (Figure 2) [110,111]. Due to the rapid development of nanotechnology in recent years, many research teams have made efforts to use nanoparticles as radiosensitizers in GBM. The tumor resensitizing effect using nanotechnology is achieved by, among other things, increasing PD-L1 and EGFR downregulation using solid lipid nanoparticles, resulting in reduced glioma growth; enhancing the EPR effect and leading to better diffusion of nanoparticles into GBM tumors; sensitizing GBM cells to radiation by bringing GBM stem cells into the radiation-sensitive G2/M phase using Sonic hedgehog ligand; increasing the number of DNA double-strand breaks using BSA-Au nanoparticles or by exposing iodine nanoparticles to radiation; and increasing BBB permeability and cellular internalization, leading to the inhibition of GBM tumor growth in vivo, using PLGA nanoparticles conjugated with chlorotoxin (CTX) [16,113]. Gold nanoparticles also appear to be effective in radiosensitization—a study by Kazmi et al. [114] showed that U87 GBM cells were radiosensitized after irradiation along with the use of AuNPs. The use of nanoparticles to silence EGFR and RELA/P65 genes associated with radiation resistance was demonstrated in a study by Cen, Bohong et al. [115], where peptide NPs carrying siRNA were tested in GBM cells. Significant gene silencing, increased apoptosis, and reduced cell viability were observed. The treatment impaired DNA repair mechanisms, thereby increasing the radiosensitivity of GBM cells. In a mouse model of GBM, the combination of nanoparticle treatment with radiation therapy significantly prolonged survival with no apparent toxicity. Nanoparticles particularly useful in enhancing the efficacy of radiotherapy are so-called high-Z metal nanoparticles, which include NPs of gold, gadolinium, silver, bismuth, and other metal oxides. They are characterized by their ability to physically amplify the radiation dose in the tissue, thereby increasing its effect. This is due to the fact that high-Z metal has higher radiation retention power than soft tissue and can induce higher energy deposition in tumor tissue [110]. These and other examples of radiosensitizing NPs touched upon in earlier paragraphs point to the importance of nanotechnology in overcoming the big obstacle in GBM treatment, which is resistance to radiotherapy. In addition to the described advantages of nanoparticles for GBM radiosensitization, such as targeted accumulation in tumor cells as in the case of PLGA nanoparticles conjugated with chlorotoxin (CTX), increasing the effect of radiation dose as in the case of high-Z metal nanoparticles, or affecting the molecular basis of radioresistance, such as DNA repair mechanisms or bringing tumor cells into a radiosensitive stage of the cell cycle, it is worth mentioning some limitations of this type of therapy [110,113,114,115]. These include the difficult-to-predict distant potential effects of treatment, due to the fact that nanotechnologies are a relatively new field in cancer therapy. For this reason, there is a risk of long-term toxicity and the accumulation of drug-conjugated nanoparticles in tissues. In addition, clinical trials are needed to estimate therapeutic, patient-safe doses. Other limitations may include the occasional difficulty of NPs in penetrating the blood–brain barrier or possibly inducing immune reactions, as well as tissue toxicity in the case of ineffective selectivity in NP accumulation in cancer cells [110,111,112,113,114,115]. These limitations should provide a direction for future research into the application of nanotechnologies in GBM therapy, primarily to prove their safety and assess the risk of side effects. Other interesting potential avenues of development may involve so-called personalized nanomedicine, which means tailoring nanoparticle therapies based on patient-specific GBM molecular profiles; Stimuli-Responsive Systems, based on smart nanoparticles that release their cargo in response to pH, temperature, or radiation; and using AI or machine learning to optimize nanoparticle formulation and predict treatment response.

## 7. Summary

Nanoparticles are very attractive therapeutic tools due to their distinctive characteristics. By using a new technology, we can work on including more accurate drugs in pharmacy to treat many untreated diseases. The new drug delivery, improved bioavailability, and enhanced targeted therapy can be the hope for today’s medicine. The use of nanotechnology represents a great advance in the treatment of glioblastoma multiforme, offering new opportunities for targeted drug delivery, the modulation of the tumor microenvironment, and enhancing the effectiveness of chemotherapy and radiotherapy. Each group of nanoparticles, organic and inorganic, has its own unique properties and is used in different ways, but what they have in common is the ability to penetrate the blood–brain barrier, high biocompatibility, and the possibility for modifications such as adding targeting ligands to their surface, encapsulating chemotherapeutics, or transferring RNA into tumor cells. Multifunctional strategies show the great potential of nanotechnology in overcoming the limitations of traditional GBM therapy, representing a promising direction toward more precise, effective, and less toxic treatment of brain tumors, especially by supporting immunotherapy and radiotherapy.

## Figures and Tables

**Table 1:** Types of liposomal NPs currently undergoing clinical trials with the status “Recruiting/not yet recruiting”. Source: ClinicalTrials.gov.

Type of NP Under the Clinical Trial	Description of the NP	Target Patients’ Group	ClinicalTrials.gov ID	Aim of the Study	Estimated Primary Completion
RNA–lipid particle (RNA-LP) vaccine.	Autologous total tumor mRNA and pp65 full-length lysosomal-associated membrane protein (LAMP) mRNA loaded DOTAP liposome vaccine administered intravenously	Adult patients with newly diagnosed GBM and pediatric patients with newly diagnosed HGG (high-grade glioma)	NCT04573140	To manufacture feasibility and safety and to determine the maximum tolerated dose (MTD) of RNA-LP vaccines	September 2026
Rhenium nanoliposomes	186 rhenium nanoliposomes (186RNL) administered through a convection-enhanced delivery catheter	Patients with recurrent or progressive malignant glioma after standard surgical, radiation, and/or chemotherapy treatment	NCT01906385	To assess the safety, tolerability, and distribution of 186RNL given by convection-enhanced delivery	December 2025
Liposomal curcumin	Liposomal curcumin (LC) in combination with radiotherapy (XRT), and TMZ and adjuvant TMZ delivered intravenously	Patients with newly diagnosed high-grade malignant gliomas	NCT05768919	To assess the tolerability, safety, and efficacy of liposomal curcumin (LC) in combination with radiotherapy (RT) and temozolomide (TMZ)	February 2026
Liposomal transcrocetin (L-TC)	Liposomal transcrocetin (L-TC) added intravenously to hypofractionated radiotherapy and concomitant temozolomide followed by adjuvant temozolomide	Patients with histologically confirmed diagnosis of glioblastoma (GBM)	NCT06477939	To evaluate the efficacy and safety of adding or not adding liposomal transcrocetin (L-TC) to hypofractionated radiation therapy and concomitant temozolomide	31 December 2032

**Table 2:** Other nanoparticles for GBM treatment currently in clinical trials with the status “Recruiting”. Source: ClinicalTrials.gov.

Type of NP Under the Clinical Trial	Description of the NP	Target Patients’ Group	ClinicalTrials.gov ID	Aim of the Study	Estimated Primary Completion
RNA–lipid particle (RNA-LP) vaccines	pp65 RNA-loaded lipid particles, (Drug Product 1), RNA-loaded lipid particles, RNA-LPs (Drug Product 2)	Adult patients with recurrent glioblastoma	NCT06389591	To demonstrate the manufacturing feasibility and safety and to determine the maximum tolerated dose (MTD) of RNA-LP vaccines	December 2026
NanoTherm therapy system—a sterile suspension of iron oxide nanoparticles	A sterile suspension of iron oxide nanoparticles	Patients with recurrent GBM	NCT06271421	To evaluate the efficacy and tolerance of using the NanoTherm therapy system in recurrent GBM	February 2027
Aguix gadolinium-based nanoparticles	AGuIX (Activation and Guidance of Irradiation by X-ray) gadolinium-based nanoparticles	Patients with brain metastases at higher risk of local recurrence with radiation alone	NCT04899908	To determine whether AGuIX (Activation and Guidance of Irradiation by X-ray) gadolinium-based nanoparticles make radiation work more effectively in the treatment of patients with brain metastases that are more difficult to control with stereotactic radiation alone	February 2025
